# Costoclavicular Joint: An Osteological Study on Clavicles with Clinical Relevance

**DOI:** 10.7759/cureus.4409

**Published:** 2019-04-08

**Authors:** George K Paraskevas, Konstantinos N Koutsouflianiotis, Kalliopi Iliou, Nikolaos Syrmos, Orestis Ioannidis, George Noussios

**Affiliations:** 1 Orthopaedics, Aristotle University of Thessaloniki, Thessaloniki, GRC; 2 Internal Medicine, General Hospital of Thessaloniki "G. Gennimatas", Thessaloniki, GRC; 3 Psychiatry, Aristotle University of Thessaloniki, Thessaloniki, GRC; 4 Neurological Surgery, Aristotle University of Thessaloniki, Thessaloniki, GRC; 5 Surgery, Medical School, Aristotle University of Thessaloniki, Thessaloniki, GRC; 6 Otolaryngology, Aristotle University of Thessaloniki, Thessaloniki, GRC

**Keywords:** clavicle, costoclavicular joint, facet, costoclavicular ligament

## Abstract

Introduction

The current study aims to detect the incidence of occurrence of a morphological variant of the impression for the costoclavicular ligament, that is a faceted apophysis of the clavicle which participates in the formation of an aberrant joint, the so-called costoclavicular joint.

Methods

A material of 208 dry clavicles, 107 of right and 101 of left side derived from an osteological collection, was examined in order to detect the likely presence of facet apophysis at the clavicular area of impression for the costoclavicular ligament.

Results

We observed three cases of oval-shaped faceted apophysis, thus an incidence of 1.44%, two in right clavicles and one in left clavicle.

Conclusion

The awareness of such a rare joint, thus the costoclavicular joint, is important for the physician, since such a joint may be mistaken for occupying space lesion, induce pain when it is osteoarthritic and decrease the costoclavicular space leading to difficulties in subclavian vein catheterization.

## Introduction

As it is widely known lateral to the articular surface of the sternal end of the clavicle, at the inferior surface, there is usually a rough oval area for the attachment of the costoclavicular ligament (CCL) called “impression for the costoclavicular ligament” (ICL) [1-3]. In some occasional instances the ICL appears in the form of a smooth elevated faceted apophysis that constitutes synovial joint with a corresponding faceted apophysis on the first rib [4]. Such faceted apophysis on the clavicle has been demonstrated in an incidence of 10% by Poirier [5], 2.61% by Cave [4] and 19.47% by Rani et al. [6] in an osteological material and in 0.31% by Redlund-Johnell [7] and 2.7% by Rani et al. [8] in radiological studies. Such faceted apophysis on clavicle may be misinterpreted as a benign lesion of the clavicle, whereas the existence of the formed costoclavicular joint (CCJ) may induce pain in cases of osteoarthritic changes or induce difficulties during subclavian vein catheterization.

In the current study we attempt to document the likely presence of the faceted apophysis of the clavicle for the CCL in an osteological material, detect its morphological features and review the relative literature.

## Materials and methods

Our material composed of dry, macerated clavicles was derived from the Osteological Collection of the Department of Anatomy of Medical Faculty of University of Thessaloniki. The total number of clavicles was 208, out of which 107 were of right side and 101 of left side. Clavicles with pathologic or traumatic changes were excluded from our material. The clavicles were of unknown age and sex. The clavicles were examined for the likely presence of faceted apophysis at the area of the ICL. For that purpose, the examination was made by two independent investigators. The precise morphology of the faceted apophysis of ICL was detected, the photographs were taken by Nikon digital camera D3400 whereas the measurements of its dimensions were made by an electronic sliding vernier digital caliper Mitutoyo Co, Japan, with an accuracy to 1 millimeter.

## Results

**Figure 1:**
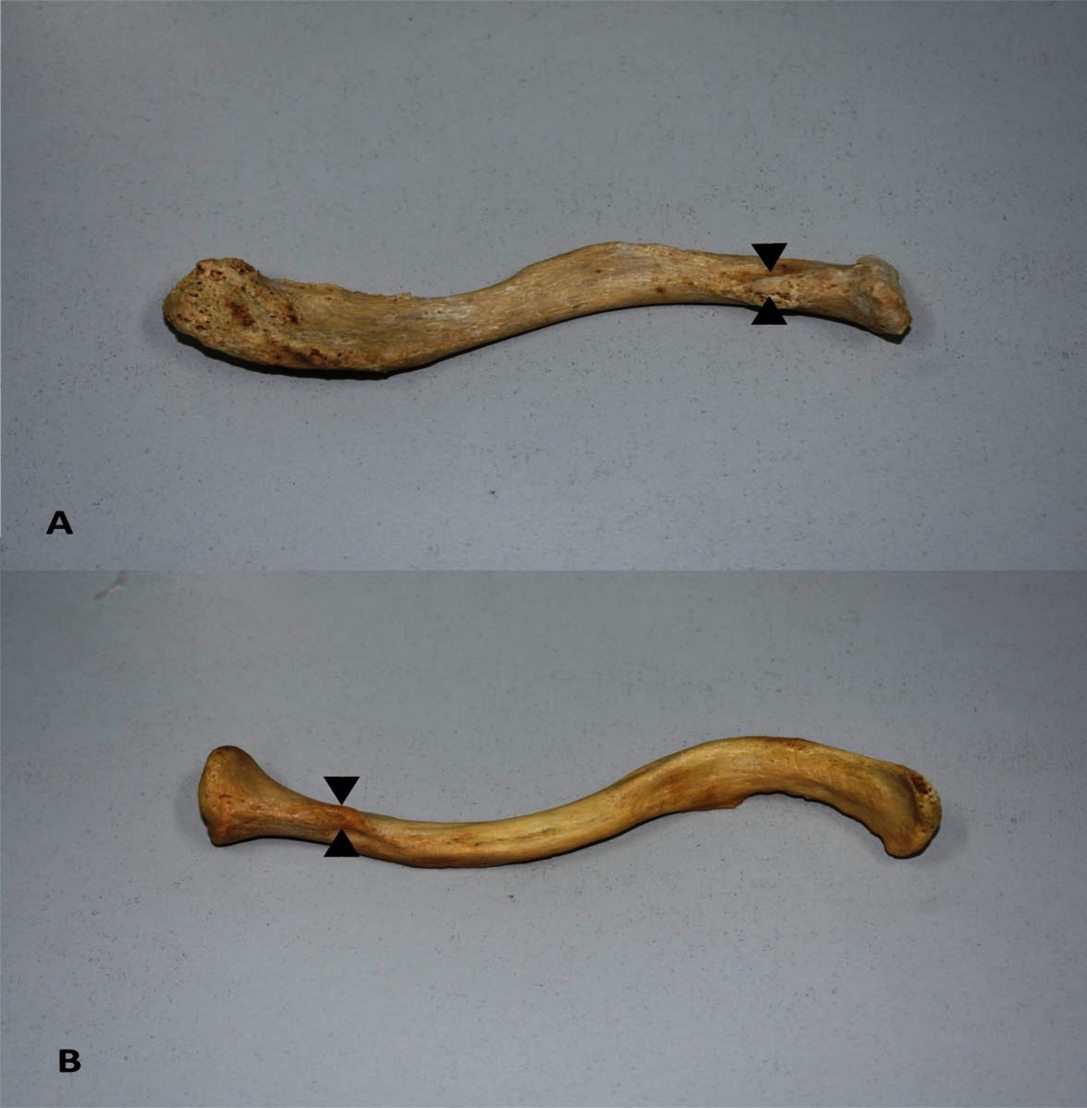
Apophyses of the impression for the costoclavicular ligament on right clavicles. The well circumscribed faceted apophyses (arrow heads) of the impression for the costoclavicular ligament of a right short (A) and right long (B) clavicle, as are shown on the inferior surface.

**Figure 2:**
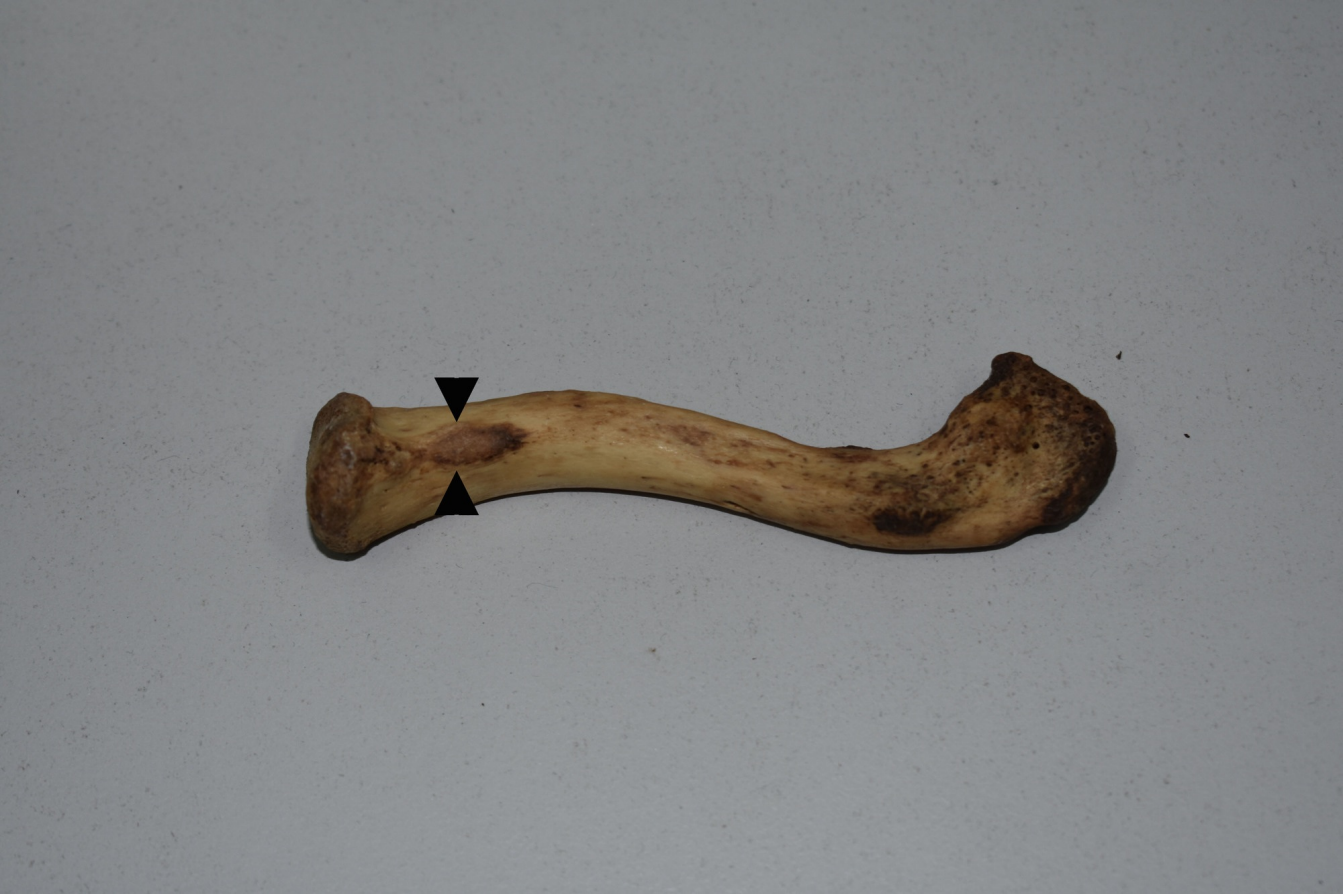
Apophysis of the impression for the costoclavicular ligament on a left clavicle. The oval-shaped faceted apophysis (arrow heads) of the impression for the costoclavicular ligament, as seen on the inferior aspect of the sternal end of a left clavicle.

Out of 208 clavicles examined, we noticed at the area of ICL that was located at the inferior aspect of the sternal end of the clavicle, three cases (1.44%) of an oval-shaped well circumscribed area of rough elevation corresponding to a faceted apophysis of an existed CCJ. In particular, in two right clavicles an oval faceted apophysis was detected with the maximum diameter being the transverse one, being 12 mm for the long right clavicle and 7 mm for the short right clavicle, whereas the transverse diameter of the faceted apophysis of the left clavicle was 11 mm (Figures 1, 2).

## Discussion

The inferior aspect of the clavicle near its sternal end is marked by a rough oval area, often depressed, for the CCL. Rarely, this area is smooth or even raised and may form a synovial joint with the first rib [2]. That area in the official anatomical terminology of Nomina Anatomica is called ICL [3]. Cave described a flat, depressed and elevated ICL in 60.78%, 29.1% and 11.1%, respectively in an osteological material [4], whereas Paraskevas et al. in a radiological study noted the above-mentioned morphological patterns in 58.12%, 26.88% and 15%, respectively [9].

The presence of aberrant diarthroses at the sternal and costal ends of the clavicle is a rare condition. The coracoclavicular joint is a diarthrosis formed between the conoid tubercle and the superior aspect of the horizontal portion of the coracoid process of the scapula, with an incidence ranging from 0.7% to 10% according to osteological studies or dissections and between 0.55% and 21% according to radiological studies [9]. As regards the CCJ, it is a synovial abnormal joint between the inferior surface of the sternal end of the clavicle and the superior aspect of the first rib or the first costal cartilage. Poirier very early in 1890 noticed in one clavicle out of 10 (10%) the existence of an ICL having the form of a smooth, elevated faceted apophysis which formed a joint with a corresponding faceted apophysis of the first rib [5]. In such cases it is considered that the CCL forms the capsule of the diarthrosis. Later in 1949 Wood Jones in Buchanan’s Manual of Anatomy mentioned an incidence of 10% for the occurrence of the CCJ, but such a statement has been considered to be merely a numerical modification of Poirier’s findings according to Cave [4, 10]. Cave in a study of 153 clavicles detected four cases, thus an incidence of 2.61%, with an elevated and smooth area of the ICL forming such abnormal diarthrosis [4]. Rani et al. in two clavicles, observed faceted apophyses for the costoclavicular and coracoclavicular joint [11]. In the study of Rani et al., a facet on the clavicle for the CCJ was documented as “elevated and smooth” and was found in a high incidence of 19.47%, whereas facets or demifacets were noted only in 9.24% on the first ribs [6]. Such a discrepancy can be explained by the fact that the CCJ may exist between clavicle and first costal cartilage only. From these facets on the rib, 8.15% showed well circumscribed area and were half oval in shape and 1.09% were oval in shape. In our study where it has been utilized the larger sample of clavicles, thus 208 in number in the literature to the best of our knowledge, the incidence of oval facets for the CCJ was 1.44%. The facets in that study were oval and well-circumscribed elevated and smooth areas. Thus, in osteological material the incidence of the faceted apophyses for the CCJ is ranged between 1.44% and 19.47%.

The first radiological documentation of the CCJ was performed by Redlund-Johnell in a total of 150 anteroposterior cervical radiographs and 500 anteroposterior radiographs of the thoracic spine; the author detected only two cases of that joint, thus an incidence of 0.31%. The joint was formed between the clavicle and the first rib [7]. Rani et al. in a series of 120 computed tomography of the cervical and thoracic region and 245 digital chest radiographs noticed 10 cases, thus an incidence of 2.7% of CCJ, detected between clavicle and ossified costal cartilage [8]. It was characteristic that the first rib did not participate in any case in the formation of the CCJ. Thus, the incidence of CCJ is ranged between 0.31% and 2.7% in radiological studies. It must be emphasized that according to Redlund-Johnell the better plain radiograph to detect a CCJ is the anteroposterior view of the thoracic spine, whereas in most cervical and chest radiographs it is difficult to detect an existing CCJ [7].

The CCL is like an inverted cone but short and flattened with anterior and posterior lamina separated by a bursa that is invariably present. These laminae are attached to the anterior and posterior lips of the ICL [1,2]. However, other authors support that CCL is a single anatomical structure [12]. It seems that CCL is a derivative of the capsule of the sternoclavicular joint and that in cases of CCJ existence, CCL forms the capsule of the diarthrosis. Potentially, the bursal cavity could be modified, forwards a synovial joint. Cave suggested that the CCJ may be developed due to wide range of clavicular movements in humans, being a next step in the evolution [4]. Rani et al. considered that the smoothness of the ICL which appears in faceted apophyses indicates the existence of bursa between the two laminae of CCL. They claimed that such a bursa can lead to formation of a synovial cavity of the CCJ [6].

Redlund-Johnell followed a patient with CCJ for 30 years and noted that this joint had not been changed, so he concluded that such a joint is congenital [7]. Osteoarthritic changes may occur in CCJ including pain in the region that should be kept in mind on behalf of the physician during the differential diagnosis of a pain arising from the sternoclavicular joint, sternocleidomastoid or major pectoral muscle. Rani et al. supported that such a CCJ can induce potentially a narrow costoclavicular space leading to difficulties during subclavian vein catheterization [8]. Furthermore, a likely CCJ should be differentiated by an osteochondroma arising from the inferior aspect of the clavicle or very rarely detected costoclavicular heterotopic ossification after severe neurological disorder [13].

## Conclusions

In our study we made an effort to detect the incidence of the impression for the costoclavicular ligament. Finally, three cases of oval-shaped faceted apophysis were found, thus an incidence of 1.44%. The knowledge of the existence of such a rare joint is important for the modern physician, since when it is osteoarthritic it induces pain and also can lead to difficulties in subclavian vein catheterization.

## References

[1] McDonald SW (1990). Last’s Anatomy: Regional and Applied, 8th Edition.

[2] Williams PL, Gray H, Bannister LH (1995). Gray’s Anatomy, 38th Edition. http://www.thegrottolibrary.info/seattle/details/3071.html.

[3] Federative Committee on Anatomical Terminology (1998). Terminologia Anatomica: International Anatomical Terminology.

[4] Cave AJE (1961). The nature and morphology of the costoclavicular ligament. J Anat.

[5] Poirier P (1890). La clavicule et ses articulations. J Anat.

[6] Rani A, Chopra J, Rani A, Raj Mishra S, Srivastava AK, Sharma PK, Dewan R (2011). A study of morphological features of attachment area of costoclavicular ligament on clavicle and first rib in Indians and its clinical relevance. Biomed Res.

[7] Redlund-Johnell I (1986). The costoclavicular joint. Skeletal Radiol.

[8] Rani A, Chopra J, Rani A, Pankaj AK, Verma RK, Dewan RK (2013). Radiological evidence of costoclavicular joint. Int J Sci Res Publ.

[9] Paraskevas G, Natsis K, Spanidou S (2009). Excavated type of rhomboid fossa of the clavicle: a radiological study. Folia Morphol.

[10] Buchanan AM (1949). Buchanan’s Manual of Anatomy, 8th Edition. https://catalogue.nla.gov.au/Record/2351238.

[11] Rani A, Mishra SR, Chopra J, Rani A, Manik P, Chauhan NK, Dewan RK (2009). Coracoclavicular and costoclavicular joints at a common juncture: a rare phenomenon. Int J Morphol.

[12] Tubbs RS, Shah NA, Sullivan BP (2009). The costoclavicular ligament revisited: a functional and anatomical study. Rom J Morphol Embryol.

[13] Lacout A, Mompoint D, Perrier Y, Vallee CA, Corlier RY (2008). Coraco- or costo-clavicular paraosteoarthropathies in patients with severe central neurological disorders. Acta Radiol.

